# Hyperbaric oxygen therapy for ischemic encephalopathy following occupational exposure to high-concentration toxic gases: two Case Reports

**DOI:** 10.3389/ftox.2026.1782213

**Published:** 2026-05-29

**Authors:** Yang Pang, Jiaojiao Yuan, Xu Yuan, Xiaomin Chai, Si Li

**Affiliations:** 1 Department of Critical Care Medicine, Hejin City People’s Hospital, Yuncheng, Shanxi, China; 2 Department of Emergency Medicine, Ninth Hospital of Xi’an, Xi’an, Shaanxi, China; 3 Department of Emergency Medicine, Hejin Traditional Chinese Medicine Hospital, Yuncheng, Shanxi, China; 4 Department of Rehabilitation, Hejin City People’s Hospital, Yuncheng, Shanxi, China; 5 Department of Emergency Medicine, Hejin City People’s Hospital, Yuncheng, Shanxi, China

**Keywords:** biogas, hydrogen sulfide gas, hyperbaric oxygen therapy, irritating gas poisoning, nervous system damage

## Abstract

Hyperbaric oxygen therapy (HBOT) is effective in managing ischemic encephalopathy of various etiologies, particularly following acute intoxication with specific gases. Considering the limited clinical evidence regarding HBOT for hypoxic encephalopathy induced by occupational exposure to toxic gases, investigating this therapeutic approach is clinically important. We report two cases of occupational poisoning due to inhalation of high-concentration toxic gases, both presenting with loss of consciousness and significant neurological deficits. Case 1 involved biogas inhalation, and case 2 involved hydrogen sulfide exposure. Following HBOT, both patients demonstrated significant neurological recovery compared to their pre-treatment status. These findings indicate that HBOT reduces neurological impairment and is associated with favorable clinical outcomes. Therefore, HBOT should be considered for ischemic encephalopathy secondary to high-risk occupational gas exposure. This approach holds potential clinical significance for preventing severe neurological sequelae in workers at risk and provides clinicians with evidence-based guidance for managing toxic gas-induced neurological injury.

## Background

Globally, high-dose toxic gas intoxication from occupational exposure remains a persistent problem, resulting predominantly from industrial accidents, equipment failures, and safety violations, particularly in confined spaces. High-risk environments include poorly ventilated confined spaces—such as drainage ditches, sewers, cellars, cesspools, and artificial digesters—where exposure to gases such as biogas and hydrogen sulfide (H_2_S) is common. Considering the wide range of chemical agents and their complex pathogenic mechanisms, the course of poisoning is determined by key factors, including gas toxicity, physicochemical properties, exposure concentration, and duration of exposure (Mackie et al., 2014).

Inhalation of high concentrations of toxic gases during occupational exposure can induce progressive pathophysiological changes, with clinical manifestations—such as respiratory dysfunction and myocardial injury—exhibiting concentration-dependent severity. In severe cases of irritant gas poisoning, patients may develop neurological abnormalities of varying intensity, including headache, impaired consciousness, seizures, and motor and coordination deficits.

Although most patients with mild acute exposure achieve complete recovery, severe poisoning—particularly in those who survive the initial critical phase—frequently results in neurological sequelae, and the most severe cases may be fatal. Hyperbaric oxygen therapy (HBOT) can mitigate tissue hypoxia, enhance microcirculatory perfusion, suppress inflammatory responses, and promote cellular repair. Both patients in this study received HBOT, which was associated with improvement in their neurological sequelae, implying a potential role for HBOT in the rehabilitation of patients with neurological injury. All case reports in this study were conducted in accordance with the case report (CARE) guidelines.

## Case presentation

### Case report 1

A 64-year-old male with a history of type 2 diabetes mellitus (T2DM) presented to the emergency department after being found unconscious in a sewer during the attempted rescue of a coworker on 2 March 2024.

The patient’s 39-year-old son had collapsed approximately 30 min after beginning sewer cleaning work and failed to respond. The patient immediately descended into the manhole in an attempt to rescue him. When both individuals remained unaccounted for, bystanders initiated a rescue operation. Unfortunately, the patient’s son was pronounced dead at the scene. The patient was admitted to the local emergency department approximately 1 h later following inhalation of an irritant gas. The exact duration of unconsciousness prior to admission could not be determined in the prehospital setting.

Upon admission, the patient presented with seizure activity. Vital signs revealed severe hypertension (blood pressure 226/136 mmHg), tachycardia (pulse 124 beats/min), tachypnea (respiratory rate 26 breaths/min), and hypoxemia (oxygen saturation 91% on 5 L/min nasal cannula); blood glucose level was elevated at 13.9 mmol/L. The patient was in moderate coma, with a Glasgow Coma Scale score (GCS) of E1V1M3, absent light reflexes with sluggish response, and bilateral positive Babinski sign. Initial laboratory evaluation revealed hepatic dysfunction (alanine aminotransferase 83.89 U/L; aspartate aminotransferase 110.32 U/L), elevated inflammatory markers (white blood cell count 17.77 × 10^9^/L; neutrophils 16.35 × 10^9^/L [91.9%]; serum procalcitonin 0.16 ng/mL), evidence of myocardial injury (serum myoglobin 135.67 ng/mL; serum troponin I 0.08 ng/mL), and hypokalemia (serum potassium 3.37 mmol/L) (Table 1). The results of metabolic acidosis testing were unavailable. Electrocardiography demonstrated nodal tachycardia, and non-contrast brain magnetic resonance imaging (MRI) with diffusion-weighted imaging revealed no abnormalities (Supplementary Appendix Picture 1). His Acute Physiology and Chronic Health Evaluation II score was 18 (Supplementary Appendix 1).

**TABLE 1:** Laboratory data.

Date	ALT/(U/L)	AST/(U/L)	TB/(umol/L)	DB/(umol/L)	Ib/(umol/L)	PH	P02/mmHg	PC02/mmHg
Day1	83.89	110.32	ND	ND	ND	7.388	95	34.9
Day2	60	32	28.65	5.39	23.26	7.48	155	39
Day3	ND	ND	ND	ND	ND	7.46	139	37
Day4	ND	ND	27.17	7.1	20.07	7.46	91	39
Day5	44	42	33.81	8.25	25.56	7.47	126	42
Day6	ND	ND	41.6	7.84	33.76	ND	ND	ND
Day7	42	29	35.08	7.56	27.56	ND	ND	ND
Day8	ND	ND	ND	ND	ND	7.47	124	42
Day9	33	25	27.6	ND	20.4	ND	ND	ND

ALT, Alanine amioTransferase; AST, aspartate aminotransferase; TB, total bilirubin; DB, direct bilirubin; IB, indirect bilirubin; ND, not detected.

The patient was administered an intravenous bolus of midazolam (5 mg) for seizure control. On post-treatment day 3, persistent impaired consciousness and inadequate sputum clearance necessitated nasotracheal intubation with mechanical ventilation, prompting initiation of HBOT. Ventilator settings were: mode, pressure-supported synchronized intermittent mandatory ventilation; fraction of inspired oxygen 40%; positive end-expiratory pressure 5 cmH_2_O; respiratory rate 12 breaths/min; and pressure support 6 cmH_2_O. Concomitant interventions included ice cap application to reduce cerebral metabolic demand, mannitol for intracranial pressure control, antibiotics for infection management, glutathione as an antioxidant, and supportive care to stabilize systemic physiology. These interventions also aimed to correct internal environmental disturbances. The HBOT protocol consisted of twice-daily sessions at 2.0 atm absolute (ATA) for 90 min during the first week, followed by once-daily sessions at 1.8 ATA for 90 min thereafter.

**FIGURE 1:**
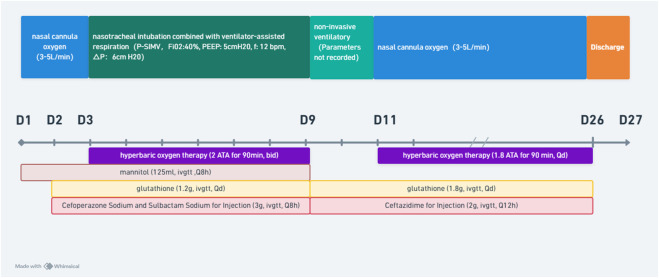
Diagnostic and therapeutic process of case 1.

At the end of post-treatment week 1, the patient’s level of consciousness improved to drowsiness (GCS = E3VTM6), with limb muscle strength graded 3/5 and persistent right-sided pathological reflexes, including a positive Babinski sign, whereas pupillary responses remained intact (Figure 1). Re-examination of the cranial CT suggested no obvious abnormalities (Supplementary Appendix Picture 2).

By day 25 of HBOT, he had regained full alertness (GCS = E4V5M6) and was independent in activities of daily living; however, residual neurological deficits persisted, including logorrhea, characterized by disinhibited speech output, and anterograde amnesia.

### Case report 2

A 27-year-old male patient experienced rapid loss of consciousness within the first minute of inhaling high-concentration H_2_S while opening a gas tank without respiratory protection. He was employed as a driver of H_2_S-containing tanker trucks and had no relevant family history of genetic disorders. Emergency transport facilitated hospital admission within the golden hour post-exposure on 8 March 2024.

Upon admission, the patient was afebrile with the following vital signs: blood pressure 155/101 mmHg, pulse 134 beats/min, respiratory rate 15 breaths/min, oxygen saturation 99% on room air, and blood glucose 11.27 mmol/L. The patient presented in moderate coma, with a GCS score of E1V1M4, intact pupillary light reflexes, and right-sided pathological reflexes, including a positive Babinski sign. Arterial blood gas analysis revealed severe metabolic acidosis with respiratory alkalosis (pH 7.009, PCO_2_ 30.4 mmHg, serum lactate > 20 mmol/L, serum bicarbonate 7.5 mmol/L, base excess ˗ 23.6 mmol/L). Laboratory evaluation demonstrated hepatic dysfunction (alanine aminotransferase 60 U/L; aspartate aminotransferase 48 U/L; total bilirubin 12.93 μmol/L; indirect bilirubin 8.12 μmol/L), elevated inflammatory markers (white blood cell count 24.48 × 10^9^/L; neutrophils 22.1 × 10^9^/L [90.3%]), evidence of myocardial injury (serum myoglobin 79.8 ng/mL; serum creatine kinase-MB 7.12 ng/mL), and hypokalemia (serum potassium 3.43 mmol/L) (Table 2). Renal function and coagulation parameters were within normal limits. Electrocardiography showed sinus tachycardia with ST-segment depression in leads V1 and V2. Brain computed tomography (CT) revealed cerebral edema (Supplementary Appendix Picture 3), and CT of the chest demonstrated aspiration pneumonia. His Acute Physiology and Chronic Health Evaluation II score was 20 (Supplementary Appendix 1).

**TABLE 2:** Laboratory data.

Date	ALT/(U/L)	AST/(U/L)	TB/(umol/L)	DB/(umol/L)	Ib/(umol/L)	PH	P02/mmHg	PC02/mmHg	Lac/(mmol/L)	HC03-/(mmol/L)	P/F/(mmHg)
Day1	60	48	12.93	4.81	8.12	7	180	30.4	>20	7.5	339
Day1	ND	ND	ND	ND	ND	7.35	202	42	2.5	23.2	404
Day2	53	32	17.3	4.61	13.14	7.32	95	46	1.9	23.7	211
Day3	36	26	ND	ND	ND	7.39	71	43	0.5	26	215
Day4	ND	ND	ND	ND	ND	7.42	80	41	0.7	26.6	242
Day6	31	21	16.2	4.01	12.19	ND	ND	ND	ND	ND	ND
Day10	37	23	7.55	2.17	5.38	ND	ND	ND	ND	ND	ND

ALT, Alanine amioTransferase; AST, aspartate aminotransferase; TB, total bilirubin; DB, direct bilirubin; IB, indirect bilirubin; LAC, lactic acid; ND, not detected.

The patient was markedly agitated and received propofol for sedation, followed by endotracheal intubation with mechanical ventilation. Concomitant interventions included ice cap application to reduce cerebral metabolic demand, mannitol for intracranial pressure control, antibiotics for infection management, glutathione as an antioxidant, and supportive care to stabilize systemic physiology. On hospital day 3, the patient’s mental status improved to drowsiness with a GCS score of E3V4M5, allowing successful extubation due to preserved spontaneous ventilation. Follow-up brain CT demonstrated persistent low-density white matter lesions (Supplementary Appendix Picture 4), suggestive of residual cerebral edema or hypoxic injury, whereas laboratory parameters gradually normalized, prompting initiation of HBOT (2.0 ATA for 90 min, two sessions/day) to facilitate neurological recovery.

By hospital day 5, the patient had fully regained consciousness, with a GCS score of E4V4M5, but continued to exhibit delayed responses to verbal and tactile stimuli. On day 7, during his first attempt at ambulation, neurological examination revealed newly developed right-sided ataxia with bilateral proprioceptive deficits, consistent with cerebellar or posterior column involvement, with a GCS score of E4V5M6.

On day 8, a 6-day course of acupuncture therapy was initiated, targeting acupoints, including Sishencong (EX-HN1), Baihui (GV20), and Jiaosun (TE20). Proprioceptive function improved, as evidenced by reduced sway on the Romberg test and enhanced ability to walk with feet together.

**FIGURE 2:**
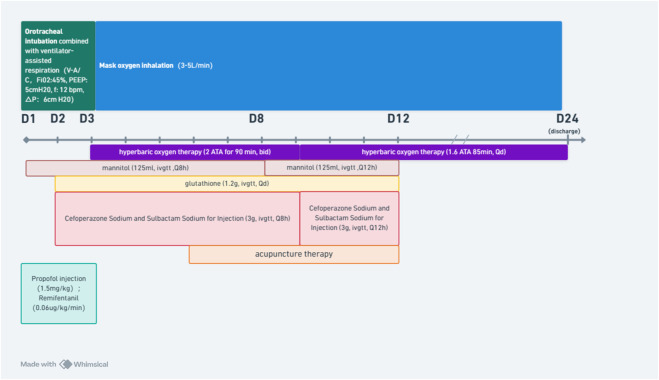
Diagnostic and therapeutic process of case 2.

On day 14, the HBOT protocol was adjusted to 1.6 ATA for 85 min, once daily. After 24 days of treatment, the patient’s neurological symptoms had resolved completely, autonomic functions had been restored, and follow-up brain MRI exhibited no abnormalities (no imaging pictures available) (Figure 2).

## Discussion

Inhalation of toxicants at concentrations exceeding occupational thresholds initiates complex toxico-dynamic processes. Acids and alkalis generated by gas dissolution directly damage cell membranes and proteins, induce protein coagulation and fat saponification, and cause cell death and tissue destruction (Squadrito et al., 2010). In addition, exposure triggers robust inflammatory and immune responses, including overexpression of tumor necrosis factor-α, interleukin-1β, and interleukin-6, as well as local infiltration of neutrophils, monocytes, and lymphocytes; these changes reduce the CD4^+^/CD8^+^ ratio and disrupt Th1/Th2 balance. Activation of transient receptor potential (TRP) channels, particularly TRPA1, TRPV1, and TRPV4, further amplifies inflammatory mediator release and immune responses, exacerbating lung injury (Zhi et al., 2007; Yali et al., 2012; Liu et al., 2018; Grace et al., 2014). Furthermore, oxidative stress is induced, leading to lipid peroxidation and depletion of antioxidant defenses, including superoxide dismutase and glutathione peroxidase (Yun et al., 2012; Gokirmak et al., 2003). In parallel, pro-apoptotic molecules, such as caspase-3 and caspase-9, are upregulated, promoting programmed cell death (Hu et al., 2018). In addition, certain gases selectively impair mitochondrial respiratory chain function, resulting in intracellular hypoxia and contributing further to tissue injury (Jiang et al., 2016).

Biogas is a mixed gas primarily composed of methane, carbon dioxide, nitrogen, hydrogen, carbon monoxide, and H_2_S. Its pathogenic effects involve methane-induced hypoxia and neurological injury from the combined gas exposure. Clinical manifestations progress through increasing severity: mild cases typically present with headache and dizziness; moderate cases are characterized by facial flushing, tachycardia, and hyperhidrosis; and severe cases exhibit critical symptoms such as profound coma, hyperthermia, tachypnea, and incontinence, which can rapidly progress to fatal respiratory paralysis if not treated promptly. Even following successful resuscitation, survivors may experience permanent sequelae, including memory impairment and psychiatric disorders (Tvedt et al., 1991a; Tvedt et al., 1991b).

​H_2_S ​is a colorless, highly toxic acidic gas and the simplest sulfur hydride, classified as an inorganic compound. At high concentrations, it paralyzes olfactory nerves, masking its characteristic odor, whereas low concentrations emit a strong rotten egg smell, and trace amounts produce a sulfur-like aroma. H_2_S irritates the eyes, respiratory system, and nervous system. Low-level exposure may cause eye irritation, headache, coughing, and dyspnea, whereas inhalation of high concentrations can be rapidly fatal (Strickland et al., 2003; Jiang et al., 2016; Reiffenstein et al., 1992; Haouzi et al., 2016). Its toxic effects involve specific inhibition of the mitochondrial electron transport chain and aerobic metabolism, resulting in intracellular asphyxia, as well as direct stimulation of carotid and aortic chemoreceptors or the respiratory center, leading to respiratory failure and death (Jiang et al., 2016; Parrish and Bradshaw, 2004; Chen et al., 2019; Yifeng et al., 2010). In addition, high-concentration H_2_S exposure can directly inhibit sodium and calcium ion channels in myocardial cells and depress the respiratory center, precipitating sudden cardiac arrest and central respiratory failure, a condition often termed “lightning death” due to its rapid lethality (Tee, 2015).​

Recent studies have confirmed that HBOT improves neurological function in patients with traumatic brain injury. Under hyperbaric conditions, the physical solubility of oxygen is significantly increased; under normal atmospheric pressure oxygen is primarily carried by hemoglobin, but HBOT elevates plasma-dissolved oxygen several-fold, enabling rapid delivery to tissue cells (Bitterman, 2009). This mechanism is particularly important for alleviating cellular hypoxia and reversing hypoxic tissue damage in situations where hemoglobin-mediated oxygen transport is compromised, such as after toxic gas exposure. In addition, HBOT reduces the production of reactive oxygen species, enhances ATPase activity, mitigates cerebral edema, and exerts neuroprotective effects (Lindenmann et al., 2010). Moreover, it lowers hematocrit and fibrinogen levels while improving red blood cell deformability, optimizing microcirculation (Leite et al., 2023; Sen and Sen, 2021). Furthermore, HBOT inhibits the release of inflammatory mediators and attenuates inflammatory cell infiltration in damaged tissues (De Wolde et al., 2021). It exerts neuroprotective effects by suppressing neuronal apoptosis through mechanisms such as regulation of anti-apoptotic gene expression and inhibition of neutrophil infiltration (De Wolde et al., 2021). Recent studies have further suggested that HBOT may promote recovery of neurological function after cerebral ischemic injury. However, its therapeutic efficacy has not been sufficiently validated because of the absence of systematic evaluation. In addition, the safety profile of HBOT remains uncertain, as some investigators have cautioned that it may precipitate airway obstruction caused by retained secretions or mucosal sloughing, posing potentially life-threatening risks (Ng et al., 2019).

Compared to the central nervous system (CNS), the peripheral nervous system (PNS) has a greater intrinsic capacity for self-repair. Injury to the CNS and PNS caused by harmful gas exposure may, over time, allow partial regeneration of the PNS under the influence of various stimulating factors, enabling partial spontaneous recovery of neurological symptoms (Lisek et al., 2024). In parallel, intensive basic nursing interventions, such as assisted limb movements and maintenance of specific postures, can exert beneficial effects on nervous system recovery. Both patients had a clear history of occupational exposure to high concentrations of toxic gases. A definitive clinical diagnosis can be established on the basis of their clinical manifestations, including presenting symptoms and neurological dysfunction, in conjunction with laboratory and imaging findings. In cases of H_2_S poisoning, mass spectrometry can be used to measure levels of the toxicant or its metabolites in urine, blood, or lung tissue to confirm the diagnosis and assess disease severity (Sastre et al., 2013; Jin et al., 2016). However, both case reports have substantive limitations, as neither patient underwent toxicological testing of biological samples, such as sulfide metabolites; this omission compromised diagnostic certainty and precluded accurate assessment of disease severity. Moreover, in cases of H_2_S poisoning, methemoglobin-forming agents, including dimethyl aminophenol, 3% nitrite, or methylene blue, can be administered as antidotal therapy; however, these treatments were not provided in our cases (Haouzi et al., 2016). Owing to the absence of toxicological confirmation, antidotal therapy was not initiated in a timely manner, which may have resulted in irreversible consequences.

Unlike prior studies, the two patients described here were in critical condition at admission and presented with severe neurological dysfunction. Following active treatment, other neurological manifestations became apparent only after improvement in symptoms, such as consciousness. During the prolonged course of HBOT, neurological symptoms did not deteriorate; ultimately, neurological function improved substantially compared to the initial stage, and no severe nervous system sequelae remained. In addition, neither patient received glucocorticoid therapy, and treatment was limited to antioxidants such as glutathione. Further clinical trials are needed to elucidate the mechanisms underlying these outcomes (van Helden et al., 2004).

Some patients who suffered from H_2_S poisoning initially showed neurological symptoms that were mainly characterized by decreased consciousness and paroxysmal motor excitement (Tvedt et al., 1991b). Over time, neurological examinations primarily revealed motor dysfunction and ataxia. A 5-year follow-up of patients who did not receive HBOT found no significant neuropsychiatric sequelae in motor function, memory, vision, or hearing. Repeat cranial MRI showed mild atrophy with no significant changes compared to previous scans. However, J. Lindenmann and colleagues found that in severe cases of H_2_S poisoning, the combination of 4-DMAP detoxification treatment and hyperbaric oxygen could restore respiratory function in victims and improve patients’ prognoses (Lindenmann et al., 2010).

## Conclusion

Neurological sequelae resulting from toxic gas poisoning are frequently overlooked, and effective therapeutic options for nervous system injury remain limited. HBOT, which increases blood oxygen tension and enhances intracellular oxygen availability, has been widely applied in the rehabilitation of hypoxic encephalopathy. In clinical practice, HBOT has been used to promote neurological function recovery in patients with cerebral infarction, carbon monoxide poisoning, and other related conditions. We report two cases of occupational poisoning due to inhalation of high-concentration toxic gases. Both patients experienced loss of consciousness and developed significant neurological deficits: case 1 involved biogas inhalation, whereas case 2 involved exposure to H_2_S. After treatment with HBOT, both patients demonstrated significant neurological recovery compared to their pre-treatment status. These findings indicate that HBOT can mitigate neurological impairment and yield favorable clinical outcomes. Accordingly, HBOT should be considered a therapeutic option for ischemic encephalopathy secondary to high-risk occupational gas intoxication. This approach has potential clinical relevance for the prevention and management of occupational gas poisoning in workers from specific industries and provides clinicians with evidence-based guidance for the treatment of neurological injury resulting from toxic gas exposure.

However, studies supporting the efficacy of HBOT for neurological injury caused by irritant gases such as biogas and H_2_S remain limited. In the two cases presented, both patients underwent prolonged courses of HBOT and ultimately achieved favorable clinical outcomes. Although the improvement in neurological symptoms cannot be attributed exclusively to HBOT, these findings imply a potential association between HBOT and neurological recovery. At present, there is no standardized consensus on HBOT protocols for patients with neurotoxicity caused by toxic gases, and the relationship between the prognosis and the severity of poisoning or treatment duration is also unclear, highlighting the need for further research.

## Data Availability

The original contributions presented in the study are included in the article/Supplementary Material, further inquiries can be directed to the corresponding authors.
