# Supplementary material for: Hyperbaric oxygen therapy for ischemic encephalopathy following occupational exposure to high-concentration toxic gases: two Case Reports
Source: Front Toxicol. 2026 May 29;8:1782213. doi: 10.3389/ftox.2026.1782213 (PMC13258541; doi:10.3389/ftox.2026.1782213)

| **Appendix 1** The APACHE II Severity of Disease Classification System | | | | | | | | | | | |
| --- | --- | --- | --- | --- | --- | --- | --- | --- | --- | --- | --- |
| PhysiologicVariable | | +4 | +3 | +2 | +1 | 0 | +1 | +2 | +3 | +4 |  |
| Temperaturerectal(℃) | | ≥41 | 39-40.9 |  | 38.5-38.9 | 36-38.4 | 34-35.9 | 32-33.9 | 30-31.9 | ≤29.9 |  |
| Mean Arterial Pressure  (mmHg) | | ≥160 | 130-159 | 110-129 |  | 70-109 |  | 50-69 |  | ≤49 |  |
| Heart Rate | | ≥180 | 140-179 | 110-139 |  | 70-109 |  | 55-69 | 40-54 | ≤39 |  |
| Respiratory Rate  (nonventilated or ventilated) | | ≥50 | 35-49 |  | 25-34 | 12-24 | 10-11 | 6-9 |  | ≤5 |  |
| Oxygenation  (mmHg) | FiO2≥0.5  use A-aDO2 | ≥500 | 350-499 | 200-349 |  | <200 |  |  |  |  |  |
|  | FiO2<0.5  use PO2 |  |  |  |  | >70 | 61-70 |  | 55-60 | ≤54 |  |
| Arterial pH | | ≥7.7 | 7.6-7.69 |  | 7.5-7.59 | 7.33-7.49 |  | 7.25-7.32 | 7.15-7.24 | <7.15 |  |
| Serum Sodium (mmol/L) | | ≥180 | 160-179 | 155-159 | 150-154 | 130-149 |  | 120-129 | 111-119 | ≤110 |  |
| Serum Potassium (mmol/L) | | ≥7 | 6-6.9 |  | 5-5.9 | 3.5-5.4 | 3-3.4 | 2.5-2.9 |  | <2.5 |  |
| Serum Creatinine  (mg/dl, Double point score  for acute renal failure) | | ≥3.5 | 2-3.4 | 1.5-1.9 |  | 0.6-1.4 |  | <0.6 |  |  |  |
| Hematocrit (%) | | ≥60 |  | 50-59.9 | 46-49.9 | 30-45.9 |  | 20-29.9 |  | <20 |  |
| White Blood Count (﹡10^9^/L) | | ≥40 |  | 20-39.9 | 15-19.9 | 3-14.9 |  | 1-2.9 |  | <1 |  |
| Glasgow-Coma-Scale (GCS) | | Score = 15 minus actual GCS | | | | | | | | | |
| Serum HCO3  (venous,mmol/L,use if no ABGs) | | ≥52 | 41-51.9 |  | 32-40.9 | 22-31.9 |  | 18-21.9 | 15-17.9 | <15 |  |
| A=Total Acute Physiology Score (APS) | | Sum of the 12 individual variable points | | | | | | | | | |
| B= Age Points | | C = Chronic Health Points | | | | | | | | | |
| ≤44years | 0 points | If the patient has a history of severe organ system insufficiency or is immunocompromised assign points as follows: | | | | | | | | | |
| 45-54years | 2points |  |  |  |  |  |  |  |  |  |  |
| 55-64years | 3 points |  |  |  |  |  |  |  |  |  |  |
| 65-74years | 5 points | 1. For nonoperative or emergency postoperative patients – 5 points | | | | | | | | | |
| ≥75years | 6 points | 1. For elective postoperative patients – 2points | | | | | | | | | |
| APACHE II Score = Sum of A (APS points) + B (Age points) + C (Chronic Health points) | | | | | | | | | | | |
| (From: Knaus WA, Draper EA, Wagner DP, Zimmerman JE. APACHE II: a severity of disease classification system. Crit Care Med. 1985 Oct;13(10):818-29.) | | | | | | | | | | | |


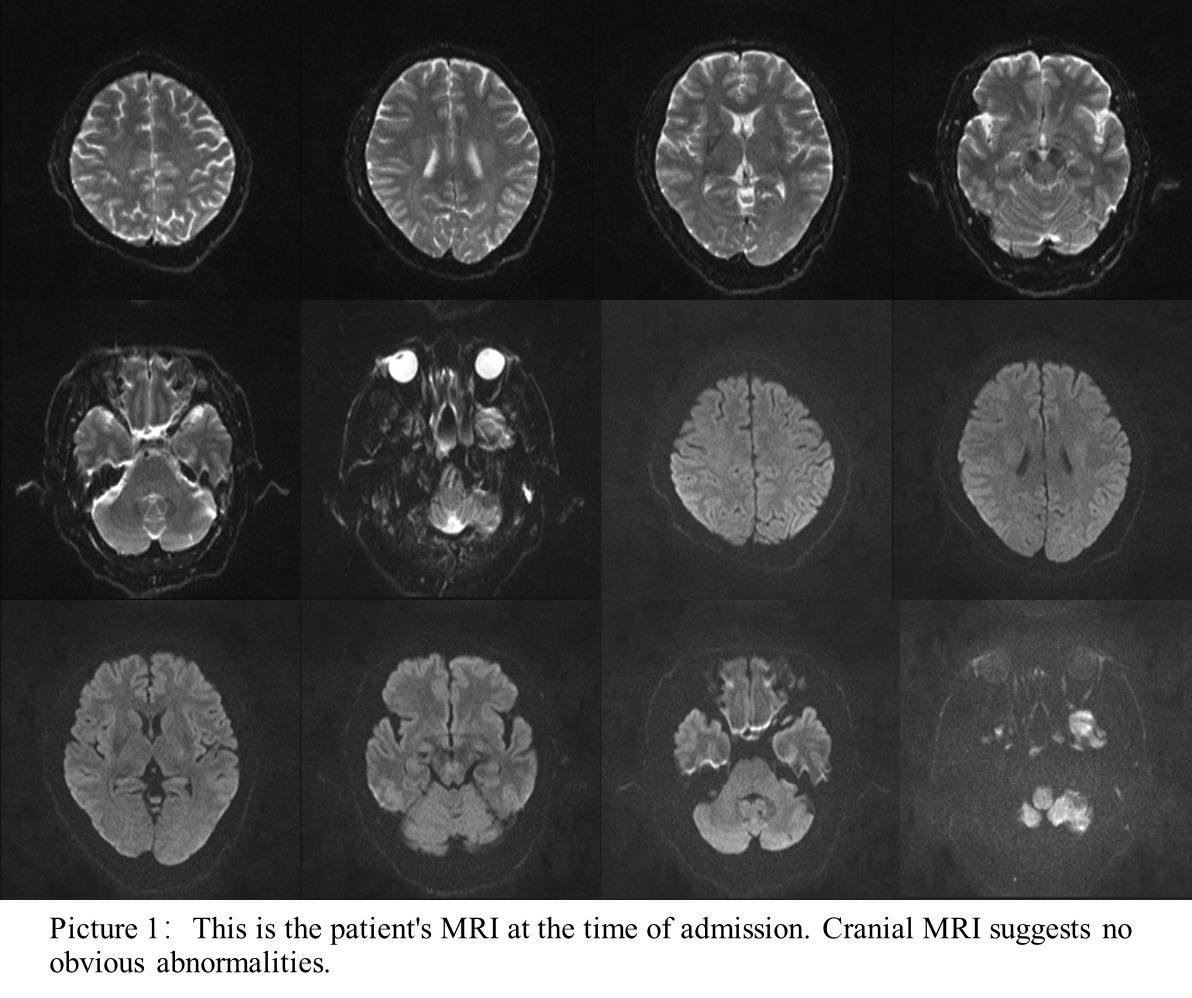

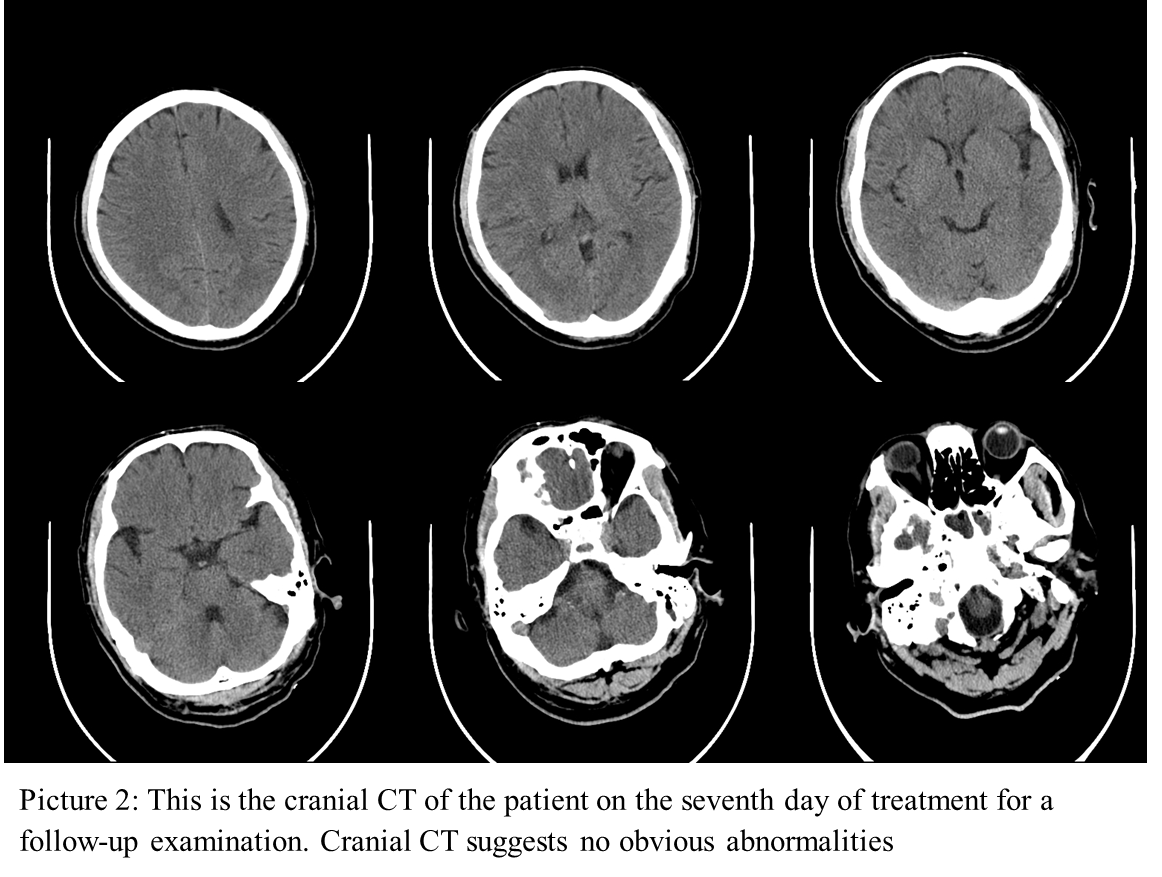

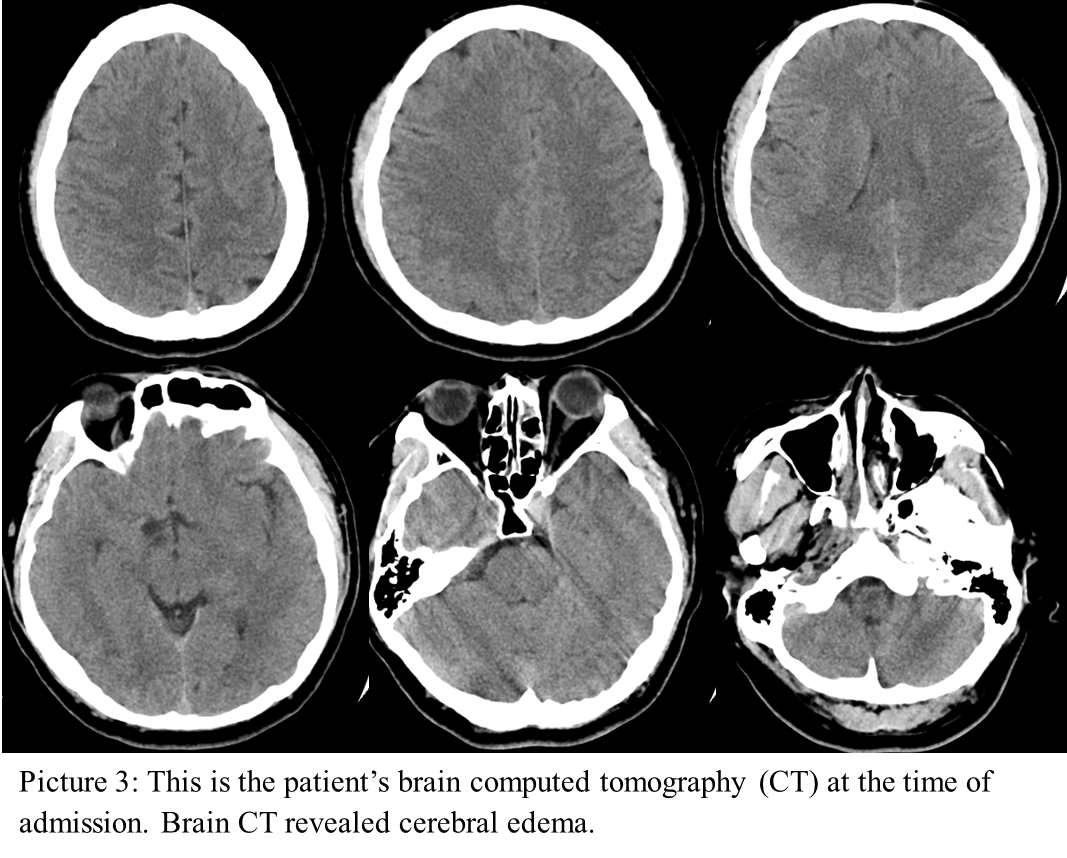

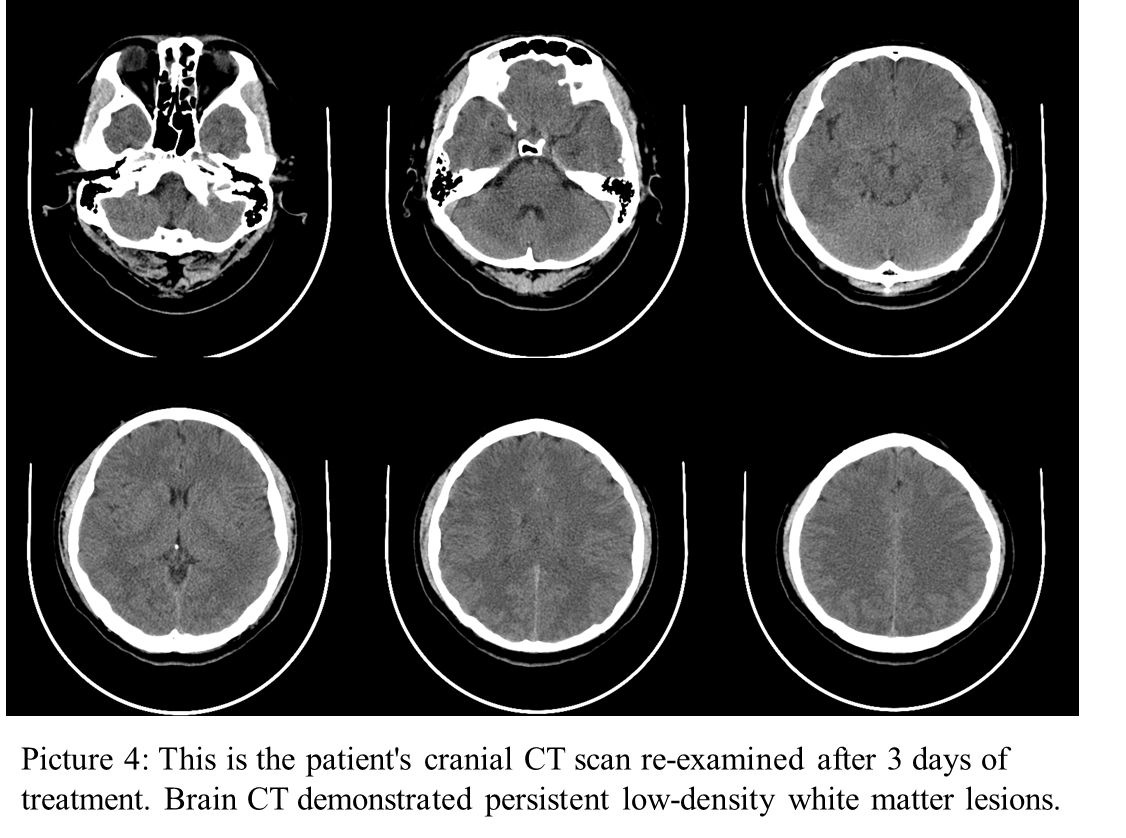

Supplement: Supplementary file 1 [file Supplementaryfile1.docx]
